# 

**Published:** 1867-01

**Authors:** 


### INDEX TO VOLUME XXI.

Alveolar Abscess 1
An Address 21
Association of the Colleges of Dentistry 29, 188
American Dental Convention 36
A New Extension Bracket 43
A Comparison of the Present with the Past 57
Address to the Graduating Class of the Ohio Dental College 89
Animal Heat.—An Inaugural Thesis 148
A Neglected Communication 152
Artificial Teeth 162
A Large Alveolar Abscess 165
A Growl from an Ignorant Man against some Scientific Technicalities 198
Absolutely Pure Gold for Filling Teeth 242
American Dental Association 274, 280, 369
A Dental Miscellany 286
Address of Dr. Geo. L. Field 304
As Funny as Ever 322
Address of W. O. Kulp, D. D. S 337
A New Dental College 370
A Random Thought on Nitrous Oxyd 371
A Replanted Tooth 453
A New Anæsthetic 534
A New Styptic 534
Baltimore College of Dental Surgery 143
Bibliographical 454
Chicago Dental Society 383
Cheap Science 366
Care of the Teeth 292
Case of Acute Trifacial Neuralgia 308

Cracking of Rubber Plates ... 249
Convention of Medical Teachers ... 278
Communications ... 230, 231
Cohesive Shred Gold ... 233
Close of the College Session ... 142
Cracking of Rubber Plates ... 38
Certain Effects of Malaria ... 475
Cleaning Teeth and Dentifrices ... 504
Cantharidal Colodion ... 526
Dental Caries Hygienically Considered ... 13
Dental Nomenclature ... 25, 41, 150
Dental Association of Canada West ... 33
Deleware Dental Association ... 36, 266
Delay ... 41
Dental Meeting ... 85, 498
Dental Education ... 86
Dr. Brown's Dental Depot ... 143
Dentinitis ... 166
Dental Teaching ... 232
Dental Instruments ... 234
Do Gum Plates Injure the Taste ... 235
Death of Dr. John McCurdy ... 273
Dental Association of Ontario ... 352
Dental Society of Western New York ... 457
Dental Chair ... 369
Duty of Dentists to Instruct their Patients Respecting the Care of the Teeth ... 381
Dental Hints ... 512
Dental Therapeutics ... 531
Extracting Teeth ... 341
Extracts from Proceedings of the Miss. Valley Dental Association 203, 251
Extracts from Discussions of the First Meeting of the Ohio State Dental Society ... 253
Enlightening and Directing Public Opinion in regard to the Duties, Responsibilities and Requirements of Dental Practitioners ... 145
Examinations for the Degree of D. D. S. in the Ohio College of Dental Surgery ... 137
Errata ... 44
Editorial ... 40, 86, 137, 185, 232, 269, 322, 364, 410, 448, 489, 537
Exostosis ... 495
Effects of Alcohol on the System ... 536
Financial ... 498
Function ... 189
Glycerine in the Arts ... 535
Histology ... 45

How to Promote the Health of the Dentist 166
Hudson River Association of Dental Surgeons 265
Hull's Benzine Gas Burner 364
How do you get along with Nitrous Oxyd ? 367
Hereditary Influences on the Teeth 405
Hermetic Seal 534
Illinois State Dental Society 435
Iowa State Dental Society 357
Improvement in Artificial Teeth 299
Indiana State Dental Association 262
In Memoriam—John S. Clark, D. D. S 186
In Memoriam—A. M. Leslie, D. D. S 143
Inquiry and Answer 17
Inherited Diseases 494, 529
Internal Use for Chloroform 536
Interesting 538
J. M. Brown 413
Liberal Prizes 185
Legal Recognition and Protection 489
Legal 537
Meetings 42
Minutes of the First Annual Meeting of the Ohio State Dental Association 67
Minutes of the Twenty-third Annual Meeting of the Miss. Valley Association of Dental Surgeons 170
Michigan Dental Association 344
Missouri Dental College 368
Mad River Dental Society 412
Management and Best Means of Preventing the Destruction of the Deciduous Teeth 425
Microscopy of the Teeth 519
Memphis Dental Society 532
Meetings of Societies 537
Notice 21, 54, 37, 43
Nitrous Oxyd Again 413
New Firm—Old Depot 414
Notice to Dentists 362
Northern Ohio Dental Association 356
Nip and Snarler 41
Nitrous Oxide 527
Old Colony Dental Association 264
Ohio State Dental Society 275, 325
"One more Unfortunate" 365
Ohio College of Dental Surgery 452
Obituary 456, 540

Organization of the Tenn. Dental Association 485
Prof. Peebles' Address 448
Personal and Unselfish 455
Principles and Practice of Filling Teeth 378
Personal 414, 41, 497
President's Address to the Miss. Valley Association, March 6, 1867 244
President's Address to the Graduates of the Ohio College of Dental Surgery 156
Proceedings of Societies 187
Reparatory 414
Red Blood Globules 281
Root of Tooth in the Antrum of Highmore 278
Response of the Class 105
Redman's Mallet Plugger 40
Replanted Teeth 493
Selfish 142
Specialties in Medicine 275
Societies 370
Saving Exposed Pulps 528
Strange Cause for Suicide 535
The Duty of the Hour 430
Tennessee Dental Association 455
The Central Ohio Dental Association 408
The Code of Ethics 410
The Relationship of Matter 327
Therapeutic 366
The Rubber Question 368
The Third Semi-annual Meeting of the Central States Dental Association 311
Teeth Extracted Without Pain 269
The Code of Ethics 271
The American Journal of Dental Science 274
The American Medical Association 279
The Canines 233
The American Dental Association 178
The Third Regular Annual Meeting of the Illinois State Dental Society 111
Transactions of the Central States Dental Association 76
The Use of the File in Dentistry 26
The Iowa State Dental Society 267
The Lake Erie Dental Association 498
Transactions of the American Dental Association, 1867 497
The Dental Association of Western New York 492
The Western New York Dental Association 484
The Preparation of Teeth for Filling 509
The Spectrum of the Crimson Tide 499

Watt's Chemical Essays 370
Western Dental Society 168
Violence in Dental Operations 39
Zinco-Magnesium Light 522

### NOTICE.

Transactions of the American Dental Association, including the proceedings of the Convention of Delegates held at Niagara Falls, August 4, 1859, and the six annual meetings held in Washington, Cleveland, Philadelphia, Niagara Falls, Chicago and Boston, in successive years, are now being published in one volume, about the size of "Harris' Principles and Practice," and containing about one hundred Essays and Addresses, with verbatim reports of the discussions. The publishing committee having received applications for copies from those not members, have decided to print what extra copies may be ordered, in addition to the number required to supply the members of the Association.

As the edition will be limited to the demand, orders from others than members (enclosing the price, $5.00,) should be forwarded immediately to the undersigned, 208 Essex St. Salem, Mass.

The books will be ready for delivery in a few weeks.

L. D. SHEPARD,
Chairman Pub. Com.

enables it to be used by hand pressure. The weight of the instrument renders it less practicable for taking up and introducing the gold, than some others.

It will necessarily be somewhat expensive, but we think after some familiarity, it would be almost invaluable in the hands of any one. Further information concerning it can be obtained by addressing Dr. W. G. Redman, Louisville, Ky.

### DELAY.

The present number of the REGISTER is, to our great regret, much delayed ; but from a combination of circumstances, that we could not prevent or control. Sickness, an unusual press of varied duties, and the —— —— printers !

### PERSONAL—PROF. WATT.

Our readers, though before notified of the fact, will be delighted to find Dr. Watt's name upon the REGISTER as one of its Editors. His editorials will bear his initial. The other Editor will get somebody to write his editorials who will consent to do so without any initial.

It is with great regret that we have occasion to announce that Dr. Watt is again for the last month prostrated by severe sickness, not, however, of as grave a character as that with which he was afflicted fifteen months ago ; but, nevertheless, very severe. We are glad to say, that within the last few days there is much improvement in his symptoms. His chair is being very satisfactorily filled in the College by Prof. Vaughn, of this city, than whom there is perhaps no man of better general scientific attainments in the country.

### NIP AND SNARLER.

### MEETINGS.

The regular annual meeting of the Ohio Dental College Association will be held in the first lecture room of the College on Tuesday the 5th day of March, at 10 o'clock A. M. As there is much and important business to be transacted, it is very desirable indeed to have a full meeting of the stockholders. The business matters of the Institution should be transacted by all the stockholders or members of the Association, and not by eight or ten persons, as has sometimes been the case.

The cause of professional education has become too important a matter to be neglected by any member of the profession who has its best interests at heart. We hope there will be a full attendance on the 5th of March.

---

The annual meeting of the Mississippi Valley Dental Society will take place in the Ohio Dental College, on Wednesday the 6th of March next, at 10 o'clock A. M., at which time we hope to meet all the old friends of this Association and the new ones too; and all who wish to become its friends. A meeting of all its children would make a large gathering. We shall hope to have a larger meeting of this body at this time, than for many years past.

It is the oldest Dental Society in existence, and there are memories linked with it that those to whom they are familiar would exceedingly regret to have fade away.

We hope to see all the members of this Society together once more. Then let all come. There are always matters of great interest before the meetings of this Society.

---

The Commencement Exercises of the twenty-first annual session of the Ohio College of Dental Surgery, will take place on Wednesday the 6th of March, at 7½ o'clock P. M., in the College. All members of the profession and friends of the Institution are cordially invited to be present. The exercises usual upon such occasions will take place.

### A NEW EXTENSION BRACKET.

### NOTICE.

The following numbers of the "Dental Recorder," and "Dental News Letter," are wanted to complete volumes of these Journals; which have been donated by the "St. Louis Dental Society," to the library of the Ohio College of Dental Surgery.

Any one having any of these numbers to donate or dispose of in any way, will please send them by mail to Dr. A. Blake, St. Louis, Mo., and if a charge is made for them, send bill.

"DENTAL RECORDER."

| | | |
|---|---|---|
| Wanted | Nos. 1 and 2.............. | Vol. I. |
| " | No. 2.................. | " II. |
| " | No. 1.................... | " IV. |
| " | No. 12.................... | " VII. |
| " | Nos. 7, 9, 10, 11, 12........ | " IX. |
| " | Nos. 5, 6, 7, 8, 10, 12..... | " X. |

"DENTAL NEWS LETTER."

Wanted Nos. 1, 2, 3, 4............ Vol. I.

### ERRATA.

In the article entitled Compensation, in the Dec. No. of the REGISTER, on page 534, third line from top, read "distribution" instead of "retribution."

And on page 535, 13th line from bottom, read "But" instead of "At." Also same page, 5th line from bottom, read "obedience" instead of "and obedience." Also on page 563, last line at bottom, for "few and many" read "pen and money." And on page 564, 11th line from top, for "runner" read "income."

### CONTENTS.

ORIGINAL COMMUNICATIONS.

Alveolar Abscess.......... 1

Dental Caries, Hygienically Considered.......... 13

Inquiry and Answer.......... 17

An Address.......... 21

Dental Nomenclature.......... 25

The Use of the File in Dentistry.......... 26

Cracking of Rubber Plates.......... 38

Violence in Dental Operations.......... 39

PROCEEDINGS OF SOCIETIES.

Association of the Colleges of Dentistry.......... 29

Dental Association of Canada West.......... 33

Delaware Dental Association.......... 36

American Dental Convention.......... 36

Notice.......... 37

EDITORIAL.

Redman's Mallet Plugger.......... 40

Delay.......... 41

Personal—Prof. Watt.......... 41

Nip and Snarler.......... 41

Meetings.......... 42

A New Extension Bracket.......... 43

Notice.......... 43

Errata.......... 44

---

### The Dental Register

OF THE WEST.

*Issued Monthly, at $3 a Year in Advance.*

DEVOTED TO THE INTERESTS OF THE DENTAL PROFESSION.

EDITED BY J. TAFT AND G. WATT.

The volume commences in January and closes in December.

The Register being issued monthly is always fresh, therefore indispensable to the practitioner who desires to keep posted up with the new inventions and improvements which are daily developed. Neither expense nor effort will be spared to give value and variety to its contents. Articles will be illustrated with well executed engravings whenever they will add to the interest or assist in explanation.

Its extensive circulation and frequency of issue makes it one of the BEST DENTAL ADVERTISING MEDIUMS in the United States.

RATES OF ADVERTISING.

One page, 1 year, $50. Half page, 1 year, $30. Quarter page, or card, 1 year $20.

All advertising bills payable in advance, or at furthest after the issue of the first number containing the advertisement. All transient advertisements to be paid for in advance.

☞ CONTRIBUTIONS SOLICITED.

Address, J. TAFT, or "DENTAL REGISTER," Cincinnati, Ohio.

Business Communications to

**J. TAFT, Publisher,**

No. 238 Fourth St., between Plum and Central Avenue.

